# Evaluating Hemodynamic Changes in Preterm Infants Using Recent YOLO Models

**DOI:** 10.3390/bioengineering12080815

**Published:** 2025-07-29

**Authors:** Li-Cheng Huang, Zi-Wei Zheng, Ming-Chih Lin, Yu-Ting Tsai

**Affiliations:** 1 Master’s Program of Electroacoustics, Feng Chia University, Taichung 40724, Taiwan; 2 Program of Mechanical and Aeronautical Engineering, Feng Chia University, Taichung 40724, Taiwan; 3 Department of Post-Baccalaureate Medicine, College of Medicine, National Chung Hsing University, Taichung 402, Taiwan; 4 Children’s Medical Center, Taichung Veterans General Hospital, Taichung 40705, Taiwan; 5 School of Medicine, National Yang Ming Chiao Tung University, Taipei 112304, Taiwan; 6 School of Medicine, Chung Shan Medical University, Taichung 40201, Taiwan

**Keywords:** echocardiography, object detection, YOLO

## Abstract

This research aims to offer a deep learning-based diagnostic approach for hemorrhagic complications linked to patent ductus arteriosus (PDA) in preterm infants. Utilizing the You Only Look Once (YOLO) algorithm, this research analyzed five key cardiac parameters derived from echocardiographic ultrasonic waves: the left ventricular ejection time (LVET), left ventricular internal dimension at diastole (LVIDd), left ventricular internal dimension at systole (LVIDs), posterior wall thickness at end-systole (HES), and RR interval between two successive R-waves. The proposed ensemble model achieved best-in-class detection accuracies for each parameter, with rates of 97.56% (LVET), 88.69% (LVIDd), 99.50% (LVIDs), 82.29% (HES), and 81.15% (RR interval). Furthermore, assessment of cardiac function using derived indices—end-systolic wall stress (ESWS) and rate-corrected mean velocity of circumferential fiber shortening (mVcfc)—achieved mean accuracy rates of 82.33% and 90.16%, respectively. This approach enables physicians to accurately evaluate cardiac function in preterm infants and facilitates the diagnosis of PDA-related hemorrhagic complications.

## 1. Introduction

### 1.1. Research Background and Literature Review

Preterm infants, especially those classified as having an extremely low birth weight (ELBW) with a weight under 1000 g or gestational age less than 28 weeks, frequently encounter significant cardiac complications after birth [1]. The abrupt transition from fetal to postnatal circulation imposes immense stress on their cardiovascular system, largely due to the sudden cessation of the low-resistance placental circulation. This interruption leads to a marked increase in systemic arterial resistance, thereby intensifying the afterload on the left ventricle [2]. Such hemodynamic challenges are further amplified in the presence of a symptomatic patent ductus arteriosus (PDA), a condition that fails to resolve spontaneously in the majority of extremely preterm infants by day seven [3]. As pulmonary vascular resistance decreases in the early days of life, a left-to-right shunt develops across the PDA, increasing pulmonary blood flow and potentially triggering complications like pulmonary edema, congestion, and, in severe cases, respiratory failure [2]. These shifts also predispose infants to adverse outcomes including intraventricular hemorrhage (IVH), necrotizing enterocolitis (NEC), bronchopulmonary dysplasia (BPD), and increased mortality [3].

The immature myocardium of preterm infants struggles to adapt to rapid changes in preload and afterload, elevating the risk of hemorrhagic events such as IVH and pulmonary hemorrhage. Understanding these hemodynamic alterations is therefore crucial for improving clinical outcomes. Echocardiographic techniques have been employed extensively to evaluate these changes. Takahashi et al. [4] demonstrated that while very-low-birth-weight infants exhibit initially lower left ventricular contractility compared to term infants, contractility normalizes by day five. Toyoshima et al. [2] proposed a tailored circulatory management approach based on the stress–velocity relationship to reduce IVH and mortality. Huang et al. [5] highlighted the efficacy of customized circulatory strategies in mitigating hemorrhagic complications associated with PDA.

Echocardiography, a non-invasive imaging modality, remains the gold standard for assessing cardiac structure and function in preterm infants [1,6]. To enhance interpretation and reduce inter-observer variability, recent research has incorporated deep learning (DL) techniques. Early applications focused on image segmentation and classification tasks, particularly for left ventricle (LV) segmentation. Moradi et al. [7] introduced MFP-U-Net, improving upon the U-Net architecture with extra convolutional layers to enhance LV segmentation accuracy. Kim et al. [6] proposed convolutional neural networks (CNNs) for simultaneously segmenting the LV endocardium and myocardium, while Liu et al. [8] employed deep pyramid local attention modules to refine structural delineation. Zhuang et al. [9] leveraged YOLOv3 to identify critical points within the ventricular chamber, aiding in segmentation tasks. Similarly, Mortada et al. [10] combined YOLOv7 with U-Net to segment the left ventricular endocardium, epicardium, and left atrium, demonstrating that integrating object detection with segmentation architectures can yield robust cardiac structural analyses.

While these studies mark significant progress, they predominantly address two-dimensional segmentation or classification tasks and rely heavily on static frames. In contrast, evaluating cardiac function in preterm infants often involves dynamic parameters extracted from motion-mode (M-mode) and pulsed-wave Doppler-mode echocardiograms. There remains a pronounced gap in the literature on applying DL-based object detection methodologies—originally advanced through large-scale benchmarks like the PASCAL VOC challenge [11]—to these time-based echocardiographic modalities.

Object detection techniques have rapidly evolved, moving from region proposal-based frameworks like R-CNN [12], Fast R-CNN [13], and Faster R-CNN [14] to single-stage detectors. Enhancements like Spatial Pyramid Pooling (SPP-net) [15] and the adoption of powerful CNN architectures including VGG [16], ResNet [17], DenseNet [18], and ResNeXt [19] have improved accuracy and efficiency. Simultaneously, lightweight architectures such as MobileNets [20], ShuffleNet [21], and SqueezeNet [22] have enabled deployment in resource-constrained environments.

On the optimization front, Adam [23] and other gradient-based improvements [24] accelerated convergence, while data pre-processing and augmentation techniques [25] enhanced model generalization. Novel activation functions like Mish [26] and specialized losses like focal loss [27] further refined detection robustness. Single-stage detectors (e.g., SSD [28], YOLO [29], YOLO9000 [30]) reframed detection as a direct regression problem, achieving near-real-time performance. YOLOV4 [31] integrated CSPNet [32], SPP modules, and Mish activation to balance speed and accuracy effectively—traits vital for medical applications that demand prompt, reliable assessments. Reviews by Zhao et al. [33] and others underscore the need to weigh computational complexity against accuracy, especially in clinical contexts.

By integrating DL-based object detection, our approach aims to automate and enhance the precision of cardiac assessments in preterm infants, ultimately improving clinical outcomes for this high-risk population. We focus on several key cardiac parameters extracted from M-mode and pulsed-wave Doppler-mode echocardiograms of preterm infants. These include the left ventricular ejection time (LVET, measured in seconds), the left ventricular internal dimension at end-diastole (LVIDd, measured in centimeters), the left ventricular internal dimension at end-systole (LVIDs, measured in centimeters), the posterior wall thickness at end-systole (HES, measured in centimeters), and the RR interval (the time between two consecutive R-wave peaks on the ECG, measured in seconds). These fundamental variables are used to compute important derived indicators such as the rate-corrected mean velocity of circumferential fiber shortening (mVcfc, measured in circumferences per second, circ/s) and end-systolic wall stress (ESWS, measured in grams per square centimeter, g/cm^2^). Automated measurement results from our system are directly compared with manual annotations by expert cardiologists, which serve as the clinical reference standard. The measurement errors observed in our deep learning approach are within the variability range previously reported for manual echocardiographic assessments [34,35], underscoring the reliability of our method as an alternative to traditional manual analysis.

In this study, we systematically compared a series of recent YOLO models, including YOLOV4, YOLOV8s [36], YOLOv10s [37], YOLOv11n/s [38], and YOLOv12n/s [39], on our clinical dataset of preterm infant echocardiograms. Meanwhile, YOLOV8’s anchor-free design and backbone enhancements achieved excellent precision in segmentation studies [36], YOLOv10 introduced NMS-free dual-assignment training for efficiency gains [37], and YOLOv11 improved depth with lightweight architectural refinements [38]. Additionally, YOLOv12 introduces attention-centric modules and hierarchical architectures [39], which, while effective in general scenes, did not translate into balanced performance on our clinical targets. To leverage these complementary strengths, we developed an ensemble framework that selects the best YOLO variant for each parameter and combines outputs via weighted fusion, resulting in the highest accuracy for derived clinical indices like the mVcfc and ESWS. 

Building on these advances, we propose employing YOLO models to detect key cardiac parameters, the LVET, LVIDd, LVIDs, HES, and RR interval—from M-mode and pulsed-wave Doppler-mode echocardiograms of preterm infants. These parameters facilitate calculation of the stress–velocity relationship—quantified by the mean velocity of circumferential fiber shortening corrected for heart rate (mVcfc) and end-systolic wall stress (ESWS). By plotting the mVcfc against ESWS, clinicians can evaluate cardiac function and select treatments accordingly [2].

**Figure 1:**
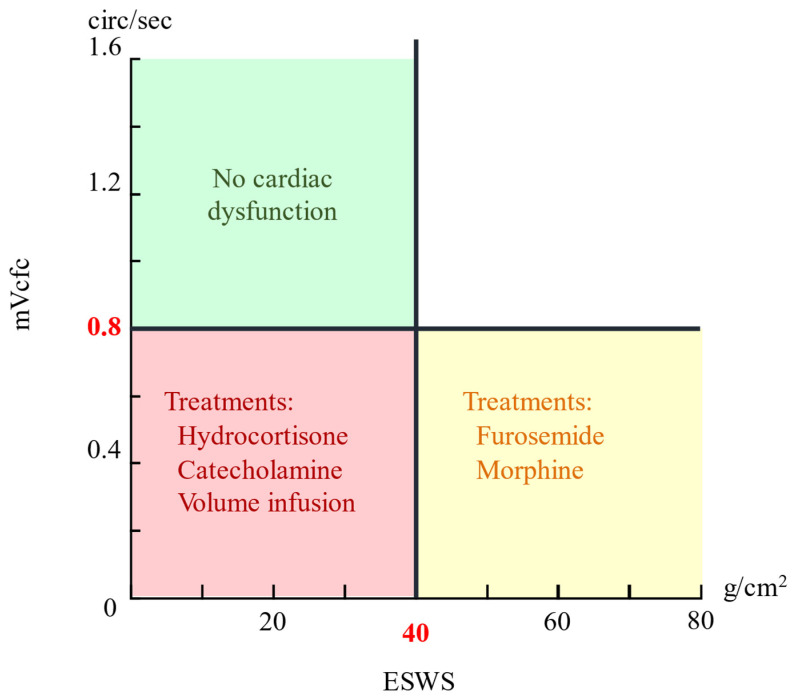
Stress–velocity relationship and treatment decision framework. Based on thresholds of mVcfc and ESWS, where mVcfc values above 0.8 circ/s and ESWS values under 40 g/cm^2^ indicate normal cardiac pump function. Treatment strategies are guided accordingly.

The formulas for the *mVcfc* and *ESWS* are given by

(1) $$mVcfc=\frac{\sqrt{RR}\cdot LVET\cdot \left(LVIDd-LVIDs\right)}{LVIDd}$$

(2) $$ESWS=\frac{1.35\cdot LVIDs\cdot MAP}{4\cdot HES\cdot \left(1-\frac{HES}{LVIDs}\right)}$$

where *MAP* is the mean arterial pressure. If the *mVcfc* > 0.8 circ/s and *ESWS* < 40 g/cm^2^, cardiac pump function is considered normal; deviations from these thresholds guide therapies ranging from hydrocortisone and volume expansion to furosemide or morphine [2]. The Cartesian coordinate system representation in Figure 1, with mVcfc and ESWS as axes, illustrates this relationship.

### 1.2. Echocardiogram Dataset of Preterm Infants

**Figure 2:**
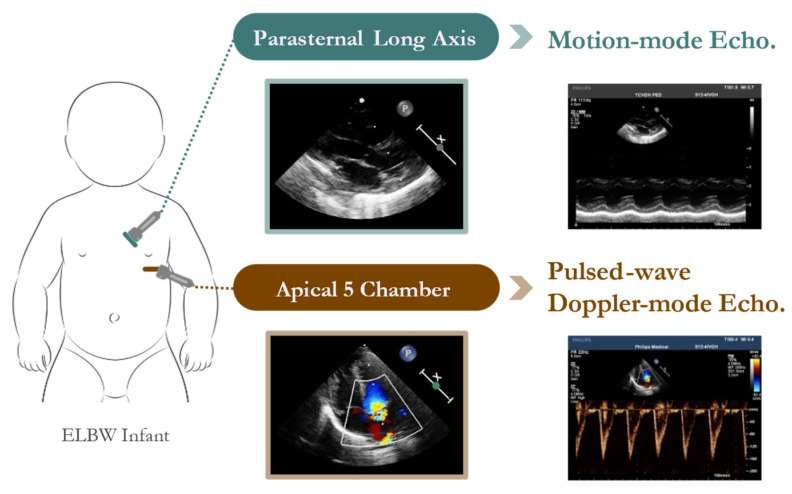
Probe placement for echocardiographic measurements in preterm infants. M-mode echocardiogram was obtained in the parasternal long-axis view. The pulsed-wave Doppler-mode echocardiographic measurements were taken in the apical five-chamber view.

The dataset analyzed in this study was obtained from the Neonatal Intensive Care Unit (NICU) at Taichung Veterans General Hospital (TVGH) in Taiwan. The echocardiographic data were acquired using a high-resolution Philips IE33 cardiovascular ultrasound system equipped with a 4 MHz sector array transducer (Philips S12-4). Standardized probe placement ensured consistent acquisition of M-mode and pulsed-wave Doppler-mode echocardiograms (Figure 2).

A total of 33 preterm infants were included, yielding 140 pulsed-wave Doppler-mode and 190 M-mode echocardiograms. Following best practices in DL-based medical image analysis, data pre-processing steps (e.g., grayscale conversion, normalization, resizing) and stochastic augmentations [25] were applied to improve model robustness and generalization. This dataset provided the foundation for training and evaluating the YOLOV4-based detectors to measure the LVET, LVIDd, LVIDs, HES, and RR interval. Leveraging the optimized architectures [17,18,22,28,29,30,32], advanced training strategies [23,24], and improved activation and loss functions [26,27], our integrated approach aims to streamline cardiac parameter detection.

By employing the latest advancements in object detection and CNN architectures [12,13,14,15,16,19,20,21,31], as well as data augmentation [25] and optimization techniques [23,24], this work bridges the gap in applying DL-based object detection to dynamic echocardiographic parameters in preterm infants. This synergy not only enhances quantitative cardiac assessments but also paves the way for more informed, timely therapeutic decisions and potentially better clinical outcomes in this fragile patient cohort.

For the LVET, our model achieves a mean percentage error (MPE) of 6.71%, corresponding to an average absolute error of approximately 2.8 ms (with a mean LVET of 41.8 ms). This level of error is within the range of inter-observer variability reported for echocardiographic timing measurements, typically 2–6% in the literature [34,35]. Previous studies have shown that manual LVET measurement by pulsed Doppler has a reproducibility error of about 4%, and most inter-observer differences are below 10% [34,35]. These results demonstrate that our automated measurement accuracy is comparable to traditional manual analysis.

## 2. Method Description

### 2.1. Data Annotation and Splitting

In this research, expert physicians and sonographers annotated cardiac parameters with rectangular bounding boxes to provide datasets for region-based object detectors. The dataset was divided into three parts for the development of the machine learning model: 60% for training, 10% for validation, and 30% for testing. The details of the dataset distribution are listed in Table 1.

**Figure 3:**
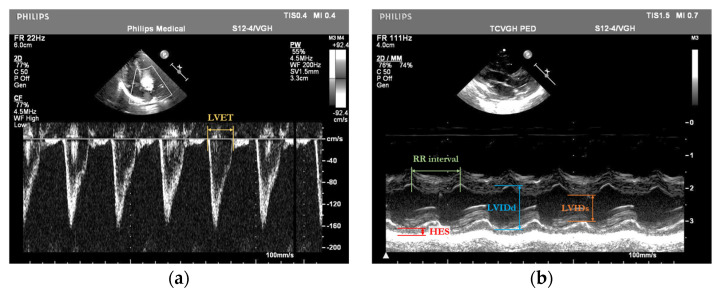
Annotation examples of (**a**) pulsed-wave Doppler-mode and (**b**) M-mode echocardiograms showing bounding boxes for LVET, LVIDd, LVIDs, HES, and RR interval.

The annotation of cardiac parameters, including the LVET, LVIDd, LVIDs, HES, and RR interval, within echocardiograms is shown in Figure 3.

### 2.2. Preprocseeing and Augmentation

#### 2.2.1. Image Cropping

To prevent information from other regions of the echocardiogram from affecting the model’s performance, the region of interest (ROI) was delineated from the echocardiogram using an image cropping technique. The ROI is defined by a 1-by-4 parameter matrix $\left[x_{min}\;y_{min}\;w\;h\right]$, where $x_{min}$ and $y_{min}$ specify the upper-left corner coordinates, w is the width, and h is the height. The echocardiograms originally measured 1024 by 768 pixels, with the specified ROIs listed in Table 2.

#### 2.2.2. Grayscale Conversion

During the pre-processing stage, grayscale conversion is applied to the echocardiographic dataset. Converting echocardiograms to grayscale ensures uniformity across the dataset.

The grayscale conversion algorithm is implemented by calculating a weighted sum of the R, G, and B components. The weightings of luminance follow the ITU-R BT.601-7 standard [40], issued by the Radiocommunication Sector of the International Telecommunication Union (ITU-R), as detailed in Table 3.

**Figure 4:**
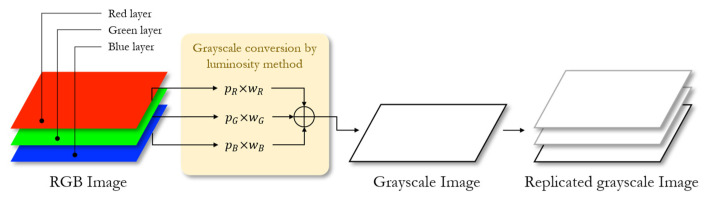
Grayscale conversion.

To match the input dimensions of the proposed object detection model, a replication operation is performed, converting two-dimensional grayscale echocardiographic images into three-dimensional data. The whole grayscale conversion progress is shown in Figure 4.

#### 2.2.3. Normalization

This procedure involves adjusting the brightness values of each pixel within echocardiographic images to a standardized scale ranging from 0 to 255, a process which is integral for uniformity.

#### 2.2.4. Rescaling

The resizing process is aimed at standardizing the size of the ROI to align with the input requirements of the network being used. In this research, the resizing dimensions were adjusted to 608 × 608 × 3 to fit the input layer of the CSPDarknet53 network.

#### 2.2.5. Augmentation

In this study, the dataset is artificially expanded through stochastic augmentations to the brightness and contrast of the echocardiograms.

The brightness adjustment is described mathematically as

(3) $$I_{bright}\left(x,y\right)=I\left(x,y\right)\times BF$$

where $I_{bright}\left(x,y\right)$ represents the pixel value after applying the brightness adjustment. This is obtained by multiplying the original pixel intensity $I\left(x,y\right)$ by a brightness factor BF. The brightness factor BF is defined as

(4) BF=0.5+γ

and it is the result of summing a base value of 0.5 with a random variable γ, which is drawn from a uniform distribution between 0 and 1:

(5) $$\gamma \sim U\left(0,1\right)$$

Correspondingly, the contrast adjustment is described mathematically as

(6) $$I_{contrast}\left(x,y\right)=\frac{I_{bright}\left(x,y\right)-min\left(I_{bright}\right)}{max\left(I_{bright}\right)-min\left(I_{bright}\right)}\times \left({CL}_{upper}-{CL}_{lower}\right)+{CL}_{lower}$$

where $I_{contrast}\left(x,y\right)$ represents the pixel value after applying the contrast adjustment. The brightness-adjusted pixel value $I_{bright}\left(x,y\right)$ is normalized. Subsequently, these values are scaled to a new contrast level, defined between ${CL}_{upper}$ and ${CL}_{lower}$:

(7) $${CL}_{upper}=0.8+\gamma_{upper}\times 0.2$$

(8) $${CL}_{lower}=0.1+\gamma_{lower}\times 0.2$$

The determination of these contrast levels is dependent on random variables $\gamma_{upper}$ and $\gamma_{lower}$:

(9) $$\gamma_{upper}\sim U\left(0,1\right)$$

(10) $$\gamma_{lower}\sim U\left(0,1\right)$$

which are drawn from a uniform distribution ranging from 0 to 1, ensuring diversity in the image contrast levels.

### 2.3. YOLO Object Detection Model

This research utilizes YOLO models, following the standard object detection framework with three primary components: the backbone, the neck, and the head. CSPDarknet53 [30] builds on Darknet53 by integrating advanced cross-stage partial (CSP) connections, significantly enhancing its efficiency and performance. The neck incorporates a Spatial Pyramid Pooling (SPP) block [ref] for multi-scale context fusion, while the head predicts at three different scales to detect objects of various sizes. The network uses a combination of convolutional, batch normalization, and activation layers (Leaky-ReLU or Mish), CSP blocks, residual units, and SPP modules to enhance feature extraction and robustness.

**Figure 5:**
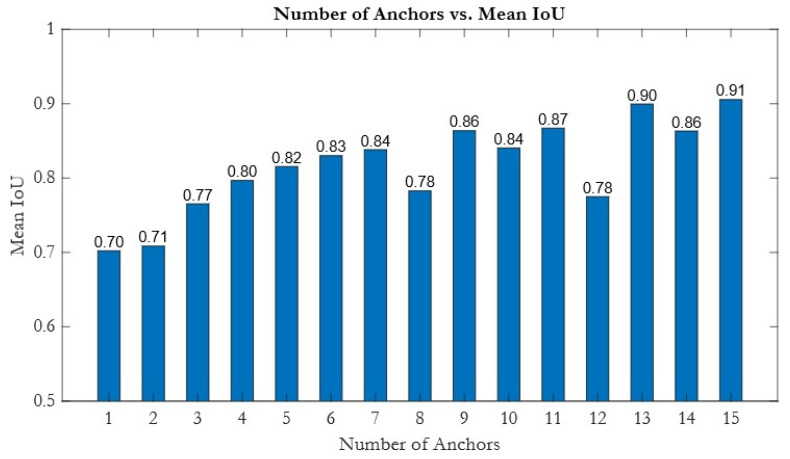
The relationship between the number of anchors and Mean Intersection over Union (IoU). As the number of anchors increases, the Mean IoU generally improves, reaching the highest value of 0.91 with 15 anchors, indicating more accurate object localization with a greater number of anchor boxes.

In the YOLO model’s anchor box design, this study systematically evaluated anchor numbers using the Mean IoU (Mean Intersection over Union) metric. The Mean IoU is calculated as the ratio of the intersection area to the union area between predicted and ground-truth boxes, averaged over all samples; it is a standard indicator for evaluating anchor coverage effectiveness. As shown in Figure 5, the Mean IoU increases significantly with the number of anchors from 1 to 7 (rising from 0.70 to 0.84), indicating that adding anchors in this range substantially improves the bounding box coverage. Increasing the anchor count to 9 further stabilizes the Mean IoU at 0.86, suggesting that more detailed anchor division can enhance the model’s generalization. When the anchor count reaches 13, the Mean IoU achieves a prominent local maximum of 0.90, indicating an optimal anchor configuration for effectively covering most ground-truth boxes. Therefore, the numbers of anchors 7, 9, and 13 in Figure 5 were chosen as candidate anchor numbers for hyperparameter optimization. Seven anchors represent a balance between accuracy and computational efficiency, nine anchors reflect further improvement with more granular coverage, and thirteen anchors correspond to the local optimum observed in the figure. Selecting these three anchor numbers as the hyperparameter scan range provides a practical trade-off between computational cost and IoU performance and serves as a foundation for further optimization of anchor sizes and aspect ratios.

The training pipeline proceeded as follows: Each YOLO detector was first pre-trained on the MS-COCO dataset and then fine-tuned on our echocardiogram dataset for 45–100 epochs, depending on the parameter (see Table 5 in Section 3.2). During fine-tuning, hyperparameter sweeps were conducted using the RMSProp, SGDM, and ADAM optimizers. The batch size was set to 16, with input images resized to 608 × 608 pixels. Early stopping based on the validation mean percentage error (MPE) was implemented to prevent overfitting and to determine the final number of training epochs for each model. Extensive data augmentation, including random brightness and contrast adjustments, was applied, as described in Section 2.2.5.

### 2.4. Post-Processing

The object detection model outputs bounding boxes representing cardiac parameters. To convert these pixel measurements into real-world values, including the LVET, LVIDd, LVIDs, HES, and RR interval, transformation ratios are applied. These ratios, based on the scale provided in the echocardiograms, allow the bounding box dimensions to be converted into physical quantities, including seconds or centimeters. The transformation ratio and unit of each cardiac parameter is shown in Table 4.

After converting the pixel measurements of the detected bounding boxes into physical values of cardiac parameters, if multiple bounding boxes for the same parameter are detected in one echocardiogram, their values are averaged using the arithmetic mean to ensure accuracy:

(11) $$x_{mean}=\frac{\sum_{i=1}^{n}x_{i}}{n}$$

where, $x_{i}$ denotes the measurement from each detected bounding box, n is the count of bounding boxes for the parameter, and $x_{mean}$ represents the computed arithmetic mean. Once the pixel measurements are converted to physical units, the cardiac parameters mVcfc and ESWS can subsequently be derived using their respective formulas.

### 2.5. Integrated Detection System

To calculate the stress–velocity relationship between the mVcfc and ESWS, five distinct YOLO models were trained for each cardiac parameter, including the LVET, LVIDd, LVIDs, LVIDs, HES, and RR interval. One YOLO model was employed for the extraction of the LVET from pulsed-wave Doppler-mode echocardiograms, while four YOLO models were individually tasked with extracting the LVIDd, LVIDs, HES, and RR interval from M-mode echocardiograms, each operating independently.

**Figure 6:**
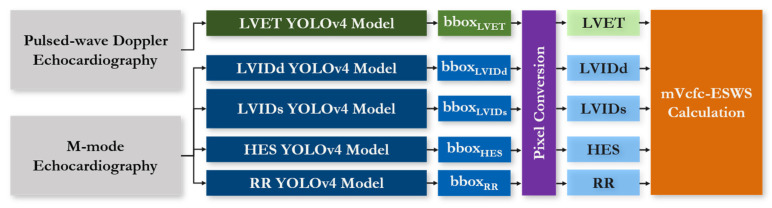
Workflow of the integrated detection system. (1) Pulsed-wave Doppler and M-mode echocardiograms are processed by separate YOLOV4 detectors for LVET, LVIDd, LVIDs, HES, and RR, each producing a bounding box for its target parameter. (2) Detected bounding box pixel measurements are converted into physical values (s, cm). (3) The converted LVET, LVIDd, LVIDs, HES, and RR values are used to compute the rate-corrected mean velocity of circumferential fiber shortening (mVcfc) and end-systolic wall stress (ESWS). (4) The computed metrics are output as clinical decision-support data.

The detected bounding boxes of each cardiac parameter were converted into physical measurements, such as centimeters and seconds, through the post-processing process. These values were then used to calculate the mVcfc and ESWS using Equations (1) and (2), providing insights into the echocardiography of preterm infants. Figure 6 shows the detection workflow of the integrated detection system.

## 3. Results and Discussion

### 3.1. Training Setup

The model training was conducted on a server equipped with an Intel^®^ Xeon^®^ Gold 5218 CPU and an NVIDIA RTX™ A6000 GPU with 48 GB onboard memory, 336 Tensor cores, and 10,752 CUDA^®^ cores. The programming environment used MATLAB^®^ R2023a with the Deep Learning Toolbox, providing a strong framework for deep learning algorithms. The system was also supported by CUDA^®^ 12.2 and NVIDIA driver version 535.54.03, ensuring compatibility and high performance for deep learning tasks.

The object detection models were initially trained using the Deep Learning Toolbox in MATLAB^®^, with each model being customized to its respective echocardiography dataset. The YOLO models were preliminarily pre-trained on the MS-COCO dataset to enhance feature extraction performance. Afterward, a transfer learning approach was used to refine the models on the echocardiography dataset, allowing for precise adaptation to the domain-specific characteristics of cardiac ultrasound images.

Based on YOLOV4 benchmarks [31] and NVIDIA RTX™ A6000 hardware by Leadtek, New Taipei City, Taiwan, the inference runs at >125 FPS for 608 × 608 images, enabling near-real-time bedside analysis.

### 3.2. Hyperparameter Optimization

The methodology used for identifying the optimal hyperparameters involves a comprehensive sweep approach. This process systematically explores a pre-defined range of hyperparameter values, enabling a comparative analysis of the resulting outcomes. Due to the extensive array of hyperparameter combinations, this process can be time-consuming. The hyperparameters examined include the number of epochs, optimizer type, learning rate, and number of anchors. The specific ranges for these hyperparameters are listed in Table 5.

**Table 5:** Hyperparameter setup for model optimization process.

Cardiac Parameters	Epoch	Optimizer	Initial Learning Rate	Number of Anchors
LVET	10	ADAM	1 × 10^−3^	6
	50	SGDM	1 × 10^−4^	12
		RMSProp		
LVIDd	50	ADAM	5 × 10^−4^	7
	75	SGDM	1 × 10^−4^	10
		RMSProp		
LVIDs	40	ADAM	1 × 10^−3^	7
	80	SGDM	5 × 10^−4^	9
		RMSProp	1 × 10^−4^	13
			1 × 10^−5^	
HES	40	ADAM	5 × 10^−3^	8
	100	SGDM	1 × 10^−4^	11
		RMSProp	1 × 10^−5^	14
RR interval	40	ADAM	1 × 10^−4^	7
	80	RMSProp	1 × 10^−5^	12

In this study, the optimization algorithms used were ADAM, Stochastic Gradient Descent with Momentum (SGDM) [24], and Root Mean Square Propagation (RMSProp). All model training and optimization procedures were performed using Ultralytics YOLO software, version 8.3.162. A detailed overview of the configuration for these optimizers is outlined in Table 6.

### 3.3. Performance Evaluation

#### 3.3.1. Evaluation Method

Traditionally, object detectors have been evaluated using standard metrics like the mean average precision (mAP), which measures the precision–recall trade-off in detection tasks. The AP is calculated by finding the area under the precision–recall curve, showing precision against recall at different confidence levels. For more specific evaluation, the AP is often calculated at various IoU thresholds, such as mAP_50_ and AP_75_, which require 50% or 75% overlap between the detected objects and ground truth. These thresholds help assess how well the detector localizes objects.

However, in this study, the cardiac parameters within echocardiograms are measured as one-dimensional physical units, such as distance and time, which are only positioned along one axis of the bounding box. If traditional evaluation methods like AP are used, it is difficult to fully capture the detection accuracy of these cardiac parameters, as the parameters represent one-dimensional distances on echocardiograms, not areas.

To better evaluate the accuracy of the detectors, the mean percentage error (MPE) was introduced. It quantifies the percentage error between the detection results and the ground truth of cardiac parameters.

For the ground truth of the test dataset, given the presence of multiple bounding boxes on each echocardiogram, the width or height of these boxes is averaged to determine the mean value of the parameter. The cardiac parameter’s mean value on an echocardiogram, $V_{GT}$, is represented as

(12) $$V_{GT}=\frac{1}{N}\sum_{j=1}^{N}V_{j}$$

where $V_{j}$ is the *j*-th ground-truth bounding box within an echocardiogram, and N is the total number of ground-truth bounding boxes.

For the bounding boxes detected by the proposed YOLO detectors, the computed mean value, $V_{det}$, is represented as

(13) $$V_{det}=\frac{1}{M}\sum_{k=1}^{M}V_{k}$$

where $V_{k}$ is the *k*-th detected bounding box within an echocardiogram, and M is the total quantity of detected bounding boxes. The *MPE* is defined as

(14) $$MPE=\frac{1}{T}\sum_{i=1}^{T}\frac{\left|V_{det}\left(i\right)-V_{GT}\left(i\right)\right|}{V_{GT}\left(i\right)}\times 100\%$$

where i refers to the *i*-th echocardiogram in the test dataset. This difference is then averaged over T, the total number of echocardiograms within the test set, to yield the overall *MPE*.

In summary, these metrics, including the AP and MPE, are employed to evaluate and compare the effectiveness and reliability of the YOLO detectors in this study.

The selection of the optimal object detectors for each cardiac parameter was based on the mAP_50_ and MPE, which are key indicators of the detectors’ accuracy. The finalized choices for the detectors, along with their tuned hyperparameters, are detailed in Table 7, as an example using YOLOV4. In this case, the LVET and LVIDd detectors achieved the highest mAP50 (92.86% and 88.69%) and lowest MPE (6.71% and 9.90%), reflecting easier detection of time-axis parameters. The LVIDs and HES demonstrate moderate performance, with mAP50 values of 68.68% and 74.36%, respectively, and MPE values of 20.18% and 25.27%, respectively. This performance can be attributed to the presence of more subtle spatial boundaries. RR interval detection (mAP50 81.2%, MPE 14.9%) falls between these groups.

**Figure 7:**
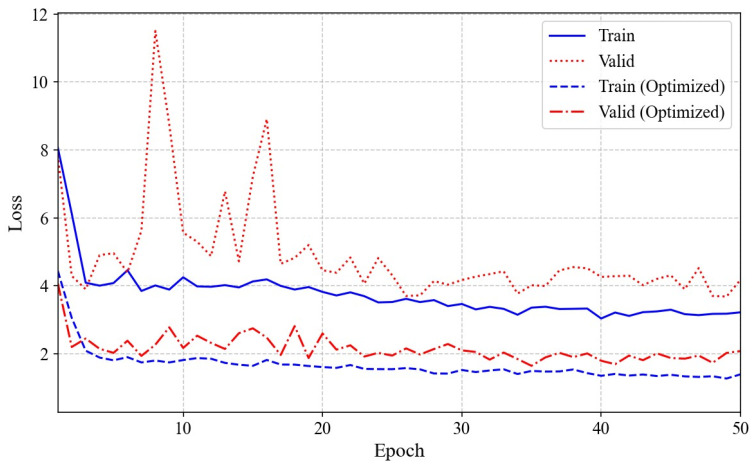
Comparison of training and validation loss curves over epochs, illustrating convergence and supporting the effectiveness of the optimal hyperparameters with an example of the LVET: the original model (blue solid line for training, red dotted line for validation) and the optimized model (blue dashed line for training, red dash–dot line for validation).

The optimal number of anchors for each cardiac parameter was determined, ranging from 6 to 15, with the highest precision achieved during optimization. RMSProp was selected as the preferred optimization algorithm due to its superior performance on the cardiac parameters. The number of training epochs for each model was determined automatically using early stopping, which monitors validation loss and halts training once performance ceases to improve. To further validate the effectiveness of our hyperparameter selection and training process, we monitored both training and validation loss across all epochs. Figure 7 presents the training and validation loss curves for both the baseline and optimized models across 50 epochs. The original model shows a gradual decrease in and stabilization of training loss, indicating effective learning of the data features. However, the validation loss remains higher and exhibits substantial fluctuations. In contrast, after applying optimization strategies such as parameter tuning, regularization, or architectural modifications, the optimized model achieves lower and more stable training losses. Notably, the validation loss for the optimized model is significantly reduced and exhibits less volatility, highlighting a marked improvement in generalization and a substantial reduction in overfitting. These results demonstrate that the adopted optimization strategies effectively enhance both the learning efficiency and the robustness of the model on validation data.

#### 3.3.2. Evaluation of mVcfc and ESWS

**Figure 8:**
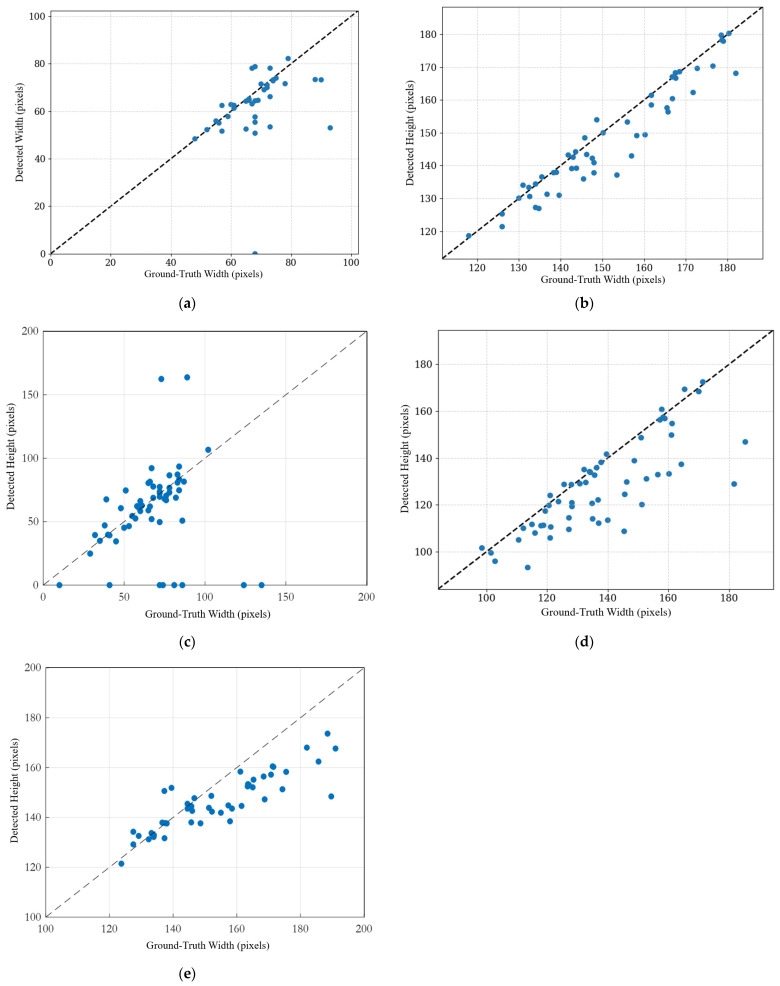
Comparison of the detection results of the YOLO model and ground-truth measurements by a human: (**a**) LVET, (**b**) LVIDd, (**c**) LVIDs, (**d**) HES, and (**e**) RR interval.

To further assess the performance of the proposed YOLO detectors, the mVcfc and ESWS were calculated using the detection results and measured MAP by Equations (1) and (2). In the assessment of cardiac performance, we define an ensemble approach that leverages the individual strengths of each YOLO model in accurately detecting specific cardiac parameters. As a result, listed in Table 8, different YOLO versions demonstrated superior performance for different cardiac parameters. We therefore selected the best-performing models regarding the mAP50 for each parameter—specifically, YOLO12s for the LVET and LVIDs, YOLOV4 for the LVIDd and RR interval, and YOLOV8s for the ESWS—and utilized their detection results to calculate the myocardial velocity of circumferential fiber shortening corrected for heart rate (mVcfc) and end-systolic wall stress (ESWS). Figure 8 presents a comparison of the detection results for each estimated cardiac parameter against the ground-truth data in pixels.

The results in Table 9 show that the ensemble approach significantly enhances the overall predictive accuracy and reliability of automated cardiac parameter extraction compared to using only YOLOV4. In addition to accurately identifying these parameters, the ensemble model notably enhanced the accuracy of clinically derived metrics, with mean accuracy rates for the mVcfc and ESWS rising to 90.16% and 82.33%, respectively. This improvement underscores the effectiveness of the ensemble method in analyzing echocardiograms, facilitating reliable and automated detection of critical cardiac indices essential for clinical decision support.

It is important to acknowledge that our study relied on a single-center dataset, which may restrict its applicability to other populations or imaging environments. We also want to underline that cases with borderline or uncertain results should still be reviewed by clinicians to ensure patient safety and accuracy. Future efforts will focus on collecting echocardiograms from multiple centers to enhance the sample size and diversity, ultimately improving the model’s generalizability and clinical utility.

## 4. Conclusions

The study demonstrated high feasibility in implementing a deep learning system that utilizes object detection techniques, particularly the YOLO architecture. This system effectively analyzes pulsed-wave Doppler mode and M-mode echocardiograms from preterm infants. The system was trained on a comprehensive dataset of echocardiographic images collected from the NICU at TVGH, Taiwan. It focuses on key cardiac parameters, including the LVET, LVIDd, LVIDs, HES, and RR interval, to compute important indicators such as the mVcfc and ESWS. By analyzing these parameters, the system provides valuable insights into the cardiac function of preterm infants, offering an evaluation based on the stress–velocity relationship. This allows medical professionals to make more informed decisions regarding the assessment of vulnerable neonates and their cardiac health.

Furthermore, innovative imaging modalities have become increasingly important in neonatal cardiology, particularly speckle-tracking echocardiography (STE). Recent studies have demonstrated that STE can effectively evaluate early changes in myocardial function after birth in preterm infants, providing insights into myocardial maturation and potential myocardial impairment [41]. Additionally, Sonaglioni et al. [42] reported that infants of gestational diabetic mothers (IGDMs) exhibit myocardial dysfunction detectable by STE, and this impairment can persist to a certain extent beyond the neonatal period. Therefore, our future studies will further evaluate the clinical application and added prognostic value of integrating traditional echocardiographic parameters and innovative STE-derived imaging features into the deep learning approach. Such multimodal integration could potentially enhance the accuracy and clinical utility of assessing cardiac function and predicting outcomes in neonates.

## Figures and Tables

**Table 1:** List of echocardiograms for model training.

Type of Echocardiogram	Data Count	Dataset Splitting		
		Training	Validation	Test
Pulsed-wave Doppler-mode	140	84	14	42
M-mode	190	114	19	57

**Table 2:** Specified ROIs on pulsed-wave Doppler-mode and M-mode echocardiograms.

Type of Echocardiogram	Specified ROIs			
	$x_{min}$	$y_{min}$	w	h
Pulsed-wave Doppler-mode	42	342	901	390
M-mode	42	350	901	382

**Table 3:** Luminance weighting of ITU-R BT.601-7 utilized in the grayscale conversion algorithm.

R-Weighting	G-Weighting	B-Weighting
0.299	0.587	0.114

**Table 4:** Units and transformation ratio of measured cardiac parameters.

Cardiac Parameter	Unit	Transformation Ratio
LVET	sec	2.653 × 10^−3^ (s/pixel)
LVIDd	cm	1.020 × 10^−3^ (cm/pixel)
LVIDs	cm	1.020 × 10^−3^ (cm/pixel)
HES	cm	1.020 × 10^−3^ (cm/pixel)
RR interval	sec	2.703 × 10^−3^ (s/pixel)

**Table 6:** Optimizer configuration for model optimization process.

Optimizer Parameter	ADAM	SGDM	RMSProp
Gradient Decay Factor	0.9	-	-
Squared Gradient Decay Factor	0.99	-	0.9
Epsilon	1 × 10^−8^	-	1 × 10^−8^
Momentum	-	0.9	-

**Table 7:** Performance evaluation of proposed YOLOV4 detectors.

Information	LVET	LVIDd	LVIDs	HES	RR Interval
Number of Anchors	6	7	9	8	12
Solver	RMSProp	RMSProp	RMSProp	RMSProp	RMSProp
Learning Rate	1 × 10^−3^	1 × 10^−4^	5 × 10^−4^	1 × 10^−4^	5 × 10^−4^
Epoch	50	75	45	100	50
mAP_50_	92.86%	88.69%	68.68%	74.36%	81.15%
MPE	6.71%	9.90%	25.27%	20.18%	14.91%

**Table 8:** Performance of YOLO versions on five cardiac parameters.

	LVET mAP50 (%)	LVIDd mAP50 (%)	LVIDs mAP50 (%)	HES mAP50 (%)	RR Interval mAP50 (%)
YOLOV4	92.86	88.69	68.68	74.36	81.15
YOLOV8s	86.96	63.35	97.65	82.29	40.95
YOLO10s	67.67	72.98	97.65	69.82	30.22
YOLO11n	88.32	55.52	99.15	67.06	40.24
YOLO11s	80.09	62.35	99.15	72.18	41.74
YOLO12n	82.51	57.85	99.50	78.91	34.22
YOLO12s	97.56	64.33	99.50	77.02	32.12

**Table 9:** Comparison of mean accuracy of mVcfc and ESWS estimations.

	mVcfc	ESWS
YOLOV4	86.14	71.83
Ensemble model	90.16	82.33

## Data Availability

The database used in this study is not publicly available. Please contact the corresponding author for access.
